# Clinical Anatomy and Measurement of the Medial Branch of the Spinal Dorsal Ramus

**DOI:** 10.1097/MD.0000000000002367

**Published:** 2015-12-31

**Authors:** Feng Shuang, Shu-Xun Hou, Jia-Liang Zhu, Yan Liu, Ying Zhou, Chun-Li Zhang, Jia-Guang Tang

**Affiliations:** From the Department of Orthopedics, The 94th Hospital of Chinese PLA, Nanchang (FS); and Department of Orthopedics, The First Affiliated Hospital of General Hospital of Chinese PLA, Beijing, China (FS, S-XH, J-LZ, YL, YZ, C-LZ, J-GT).

## Abstract

Percutaneous block and neurotomy of the medial branch of the spinal dorsal ramus has shown excellent results in treating facet joint-mediated low back pain. This study aimed to describe the clinical anatomy of the medial branch and its measurements.

We dissected the lumbar spine of 12 adult cadavers (24 sides) and measured the distances between the medial branch and various anatomical landmarks. The distances were compared between L1 and L5 vertebrae.

The distance between the dorsal ramus bifurcation and the superior border of the root of the transverse process was 3.52 ± 1.15 mm, 3.63 ± 1.36 mm, 3.46 ± 1.31 mm, 3.38 ± 1.24 mm, and 1.87 ± 0.88 for L1 to L5, respectively. The medial branch of the dorsal ramus is enclosed in a fibro-osseous canal bounded by the accessory process, the mammillary process, and the mammilloaccessory ligament.

For the percutaneous treatment of block and neurotomy, the first choice of target is the medial branch fibro-osseous canal near to the accessory process. The accessory process is not displayed in x-ray films; therefore, the junction of the superior articular process and the root of the transverse process can be targeted.

## INTRODUCTION

The lumbar facet joints are synovial joints and enable movements such as flexion, extension, and rotation of the lumbar spine. However, excessive movements of the lumbar spine may cause injury to the facet joints.^1^ The lumbar facet joints are innervated by the medial branch of the spinal dorsal ramus. The facet capsules and adjacent tissue are rich in nociceptive receptors, which causes pain when the capsules are irritated by mechanical stimulation or inflammation.^2,3^

Block of the medial branch of the spinal dorsal ramus is an effective method for the diagnosis of low back pain caused by the facet joints and is used to locate the facet joint that causes the pain. Percutaneous neurotomy of the medial branch using methods such as radiofrequency denervation has shown excellent results in treating facet joint-mediated low back pain. Neurotomy treatment has been shown to significantly improve the quality of life and reduce the dose of analgesics with short- to medium-term efficacy.^4–6^ However, the reported efficacy of different methods for radiofrequency denervation varied greatly.^5,7^ The treatment efficacy of the medial branch neurotomy lasts only ∼1 year and the response rate is low. This might be caused by inaccurate positioning of the electrode due to anatomical variation of the medial branch of the spinal dorsal ramus.

The medial branch of the dorsal ramus is enclosed in a fibro-osseous canal bounded by the accessory process, the mammillary process, and the mammilloaccessory ligament.^8^ Therefore, this fibro-osseous canal is used as the target of block and neurotomy of the medial branch. Due to the unclear image of the accessory processes in x-ray films, the junction of the lateral aspect of the superior articular process and the root of the transverse process is targeted for neurotomy of the medial branch.^8,9^ However, the accessory processes are clearly imaged using computed tomography (CT).^10^ Thus, the fibro-osseous canal is targeted for block and neurotomy of the medial branch under the CT guidance.

Using imaging navigation techniques, the needle is accurately and safely advanced to the targeted area for the treatment of low back pain caused by the facet joints. Therefore, thorough understanding of the spinal dorsal rami and its branches are important. In this study, for the purpose of percutaneous treatment of low back pain, we measured the following distances. We measured the distance between the dorsal ramus bifurcation and the superior border of the root of the transverse process; the distance between the medial branch at the root of the transverse process and the median line; the distance between the medial branch at the root of the transverse process and the body surface; the distance between the midpoint of the fibro-osseous canal and the median line; and the distance between the midpoint of the fibro-osseous canal and the body surface.

## MATERIALS AND METHODS

### Cadavers

Twelve adult cadavers (24 sides) were used in our study, including 9 men and 3 women. The mean age at death of these cadavers were 58.5 years (range 43–69 years). The cadavers had no history of spinal tumor, trauma, or skeletal disease. The cadavers were provided by the Department of Anatomy of the Southern Medical University and the Orthopedics Institution of the PLA General Hospital. This study was approved by the Ethics Committee of the Southern Medical University.

### Dissection of the Cadavers

**FIGURE 1:**
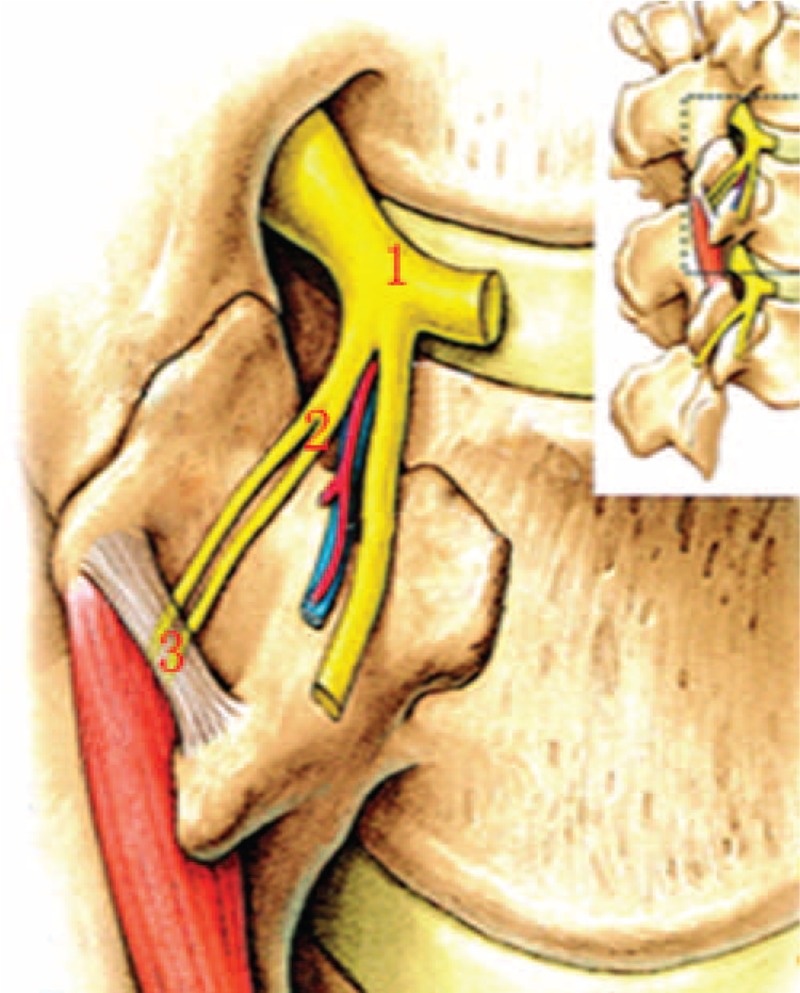
Measurement of the spinal dorsal ramus. (1) The bifurcation of the dorsal ramus into the medial and the lateral branches. (2) The medial branch at the root of the transverse process. (3) The midpoint of the fibro-osseous canal.

The cadaver was put in the prone position. An incision was carried out along the posterior median line. The medial and lateral branches of the spinal dorsal rami were exposed using blunt dissection. The articular processes, mammillary processes, and the roots of the transverse processes were recognized as anatomical landmarks. The course of the medial branch and the fibro-osseous canal were dissected and observed. We measured the distance between the dorsal ramus bifurcation and the superior border of the root of the transverse process; the distance between the medial branch at the root of the transverse process and the median line; the distance between the medial branch at the root of the transverse process and the body surface; the distance between the midpoint of the fibro-osseous canal and the median line; and the distance between the midpoint of the fibro-osseous canal and the body surface (Fig. 1).

### Statistical Analysis

All data were presented as mean ± standard deviation. Comparisons were made using the ANOVA analysis followed by the *q* test. Statistical analyses were performed using the SPSS 17.0 software (SPSS, Chicago, IL). *P* < 0.05 was considered statistically significant.

## RESULTS

### Branches of the Dorsal Rami

**FIGURE 2:**
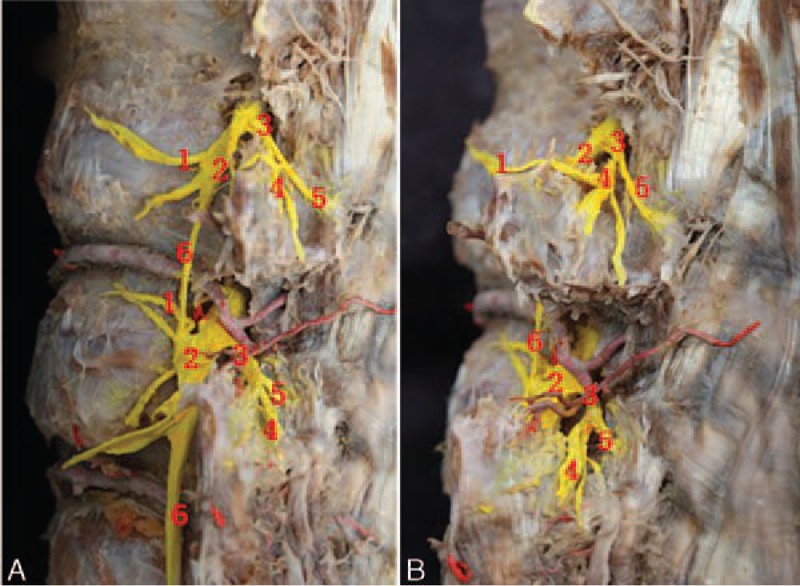
Branches of the spinal dorsal ramus. (A) Lateral view. (B) Oblique view. (1) Communicating branch of the spinal nerve. (2) Ventral ramus. (3) Dorsal ramus. (4) Lateral branch of the dorsal ramus. (5) Medial branch of the dorsal ramus. (6) Communicating plexus.

The spinal nerve exits the intervertebral foremen and divides into the meningeal branch, communicating branch, ventral ramus, and dorsal ramus. The dorsal ramus is a small branch and runs dorsally on the medial aspect of the intertransverse muscles. The dorsal ramus divides into the medial branch and the lateral branch, forming a 30-degree angle at the superior border of the lower transverse process. The medial and lateral branches of the dorsal rami supply the surrounding skin and muscles. Most of the dorsal rami show a segmental distribution pattern. Rich anastomoses are seen between the adjacent medial branches, lateral branches, and dorsal rami (Fig. 2).

**FIGURE 3:**
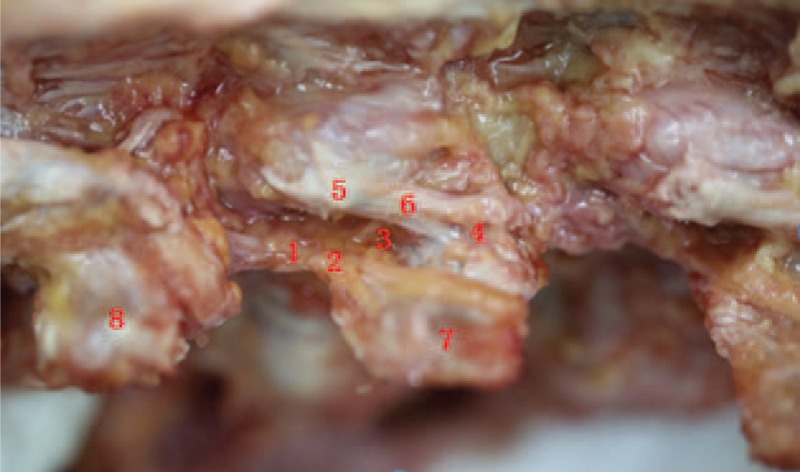
The spinal dorsal ramus and the fibro-osseous canal. (1) Dorsal ramus. (2) Lateral branch of the dorsal ramus. (3) Medial branch of the dorsal ramus. (4) Mammillary process. (5) Accessary process. (6) Mammilloaccessory ligament. (7) L2 transverse process. (8) L1 transverse process.

The medial branch runs posteriorly, medially, and caudally at the lateral border of the superior articular process of the lower vertebra. The medial branch then runs around the lateral border of the superior articular process, tightly adhering to the dorsal aspect of the root of the lower transverse process. Then the medial branch enters into the fibro-osseous canal bounded by the accessory process, the mammillary process, and the mammilloaccessory ligament. After exiting the fibro-osseous canal, the medial branch descends medially and caudally along the lamina, innervating the structures medial to the facet joint line. The medial branch also gives rise to small articular branches both before and in the fibro-osseous canal, supplies the lateral and inferior aspects of the lumbar facet joints. After exiting the fibro-osseous canal, the medial branch gives rise to small branches, innervating the medial and superior aspects of the facet joints. Therefore, 1 facet joint is supplied by the medial branches from 2 adjacent dorsal rami. The L1–3 medial branches descend 1–2 vertebrae, and the L4–5 medial branches descend 2–3 vertebrae. These medial branches penetrate the deep fascia near the median line to the subcutaneous tissue, and innervate the facet joints, the multifidus muscles, the interspinous ligament, and the supraspinous ligament. The L4–5 medial branches reach to the dorsal aspect of the sacrum and supply the lumbosacral joint (Fig. 3).

### Distance Between the Dorsal Ramus Bifurcation and the Superior Border of the Root of the Transverse Process

The dorsal ramus divides into the medial and the lateral branches at the superior border of the transverse process of the lower vertebra. The distance between the dorsal ramus bifurcation and the superior border of the root of the transverse process was 3.52 ± 1.15 mm, 3.63 ± 1.36 mm, 3.46 ± 1.31 mm, 3.38 ± 1.24 mm, and 1.87 ± 0.88 for L1 to L5, respectively. The L5 distance was significantly shorter than the L1–4 distances (*P* < 0.01). However, there was no significant difference among L1–4 distances.

### Distance Between the Root of the Transverse Process and the Median Line/Body Surface

The medial branch of the dorsal ramus runs on the lateral aspect of the superior articular process, tightly adhering to the dorsal aspect of the transverse process of the lower vertebra. The distances between the junction of the medial branch and the root of the transverse process and the median line/body surface are listed in Table 1. There were significant differences between the L1–5 median line distances except between the L3 and L4 distances (*P* < 0.01). There were significant differences between the L1–5 body surface distances except between the L2 and L3 distance (*P* < 0.01).

**TABLE 1:**
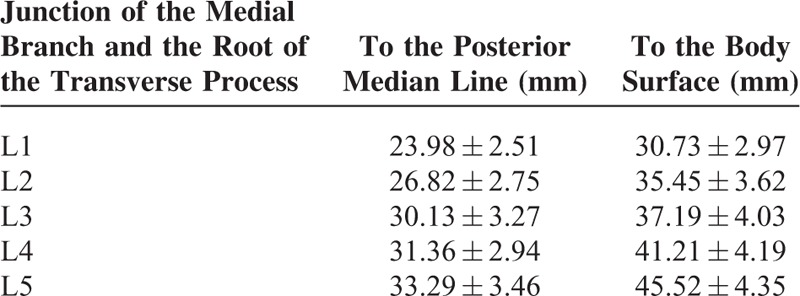
The Distances Between the Junction of the Medial Branch and the Root of the Transverse Process and the Median Line/Body Surface (n = 24)

### Distance Between the Midpoint of the Fibro-Osseous Canal and the Median Line/Body Surface

The medial branch of the dorsal ramus is enclosed in a fibro-osseous canal bounded by the accessory process, the mammillary process, and the mammilloaccessory ligament. The distances between the midpoint of the fibro-osseous canal and the posterior median line/body surface are listed in Table 2. There were significant differences between the L1–5 median line distances except between the L2 and L3 distances (*P* < 0.01). There were significant differences between the L1–5 body surfaces (*P* < 0.01).

**TABLE 2:**
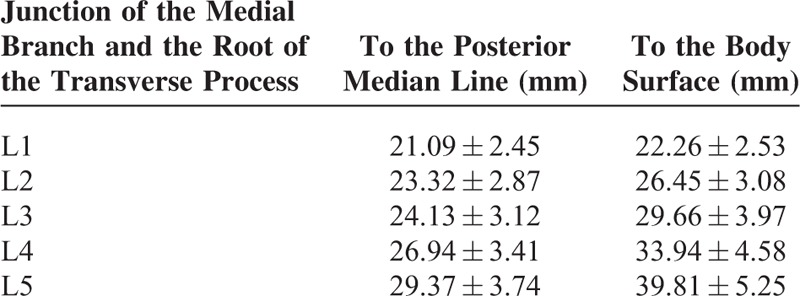
The Distances Between the Midpoint of the Fibro-Osseous Canal and the Posterior Median Line/Body Surface (n = 24)

## DISCUSSION

The lumbar zygapophysial joints, also known as the lumbar facet joints, are composed of the adjacent articular processes. Pathological changes of the facet joints are important reasons for low back pain, accounting for 15 to 40% of all etiologies.^11^ Repeated injury and trauma may cause dislocation of the spine and facet joints, retracting or compressing the medial or lateral branches of the dorsal rami, leading to low back pain.

Some authors found that the dorsal ramus divides into the medial branch, the lateral branch, and the middle branch. The middle branch arises directly from the dorsal ramus, or shares a common short trunk with the lateral branch.^9,12^ Bogduk et al also noticed that the L5 dorsal ramus only divided in 2 branches, lacking the lateral branch.^9,12^ However, the mainstay view is that the dorsal ramus divides into the medial and the lateral branches.^10,13^ We confirmed the existence of the middle branch of the dorsal ramus, which connects to the lateral branch via communicating plexus. Some previous studies might recognize the middle branch as a sub-branch of the lateral branch. Generally, it is acceptable to divide the dorsal ramus into the medial and the lateral branches.

The dorsal ramus gives rise to the lateral and the medial branches on the superior border of the transverse process. The lateral branch runs laterally, posteriorly, and caudally on the inferior aspect of the lower transverse process, innervating the skin, muscles, and ligaments lateral to the facet joint line. The medial branch runs in the groove formed by the lower transverse process and the superior articular process, and then descends caudally and posteriorly, accompanying the vessels arising from the lumbar artery and vein. The medial branch then runs around the lateral border of the superior articular process and enters the fibro-osseous canal. The L5 dorsal ramus runs on the bottom of the groove formed by the S1 superior articular process and the sacral ala, giving rise to the lateral and the medial branches. The medial branch runs medially and around the lateral aspect of the lumbosacral facet joint. The lateral branch descends and merges into the S1 dorsal ramus.^10^ The medial branch then innervates the capsules of the lower 2 to 3 facet joints, and the spine, skin, muscles, and ligaments medial to the facet joint line. The paraspinal multifidus muscles are critical for the lumbosacral stability and are innervated solely by the medial branch of the dorsal ramus, which is of special clinical significance.

The medial branch of the dorsal ramus runs through the fibro-osseous canal. The fibro-osseous canal lies on the dorsal aspect of the superior articular process and the root of the transverse process, in the bony groove formed by the mammillary process and the accessory process, running in an oblique direction. The fibro-osseous canal is bounded by 4 walls, including the mammillary process as the superior wall, the accessory process as the inferior wall, the groove between the mammillary process and the accessory process as the anterior wall, and the ligament between the superior articular process and the accessory process as the posterior wall. It has been shown that the medial branch in the fibro-osseous canal is near to the medial aspect of the accessory process rather than the lateral aspect of the mammillary process.^10^ We found that the medial branch in the fibro-osseous canal is more near to the accessory process. For the convenience, we measured the distance from the midpoint of the mammilloaccessory ligament. The mammilloaccessory ligament is a part of the medial side of the intertransverse ligament and has a tendency of ossification, forming a bony bridge between the accessory process and the mammillary process, which makes the fibro-osseous canal a completely bony tunnel. However, we did not notice any ossification of the mammilloaccessory ligament in our study. This might be attributed to the relative young age of death of our cadavers and the small sample size.

Low back pain caused by the lumbar facet joints can be treated with block or neurotomy of the medial branch of the dorsal ramus, such as percutaneous ablation, cryotherapy, laser, and surgery.^7,14–17^ Precise location of the medial branch is critical for successful treatment. The percutaneous block and neurotomy of the medial branch are usually performed under the guidance of C-arm x-ray or CT. The medial branch runs through the fibro-osseous canal and is more near to the accessory process. Therefore, the optimal target is the accessory process side of the fibro-osseous canal. However, the accessory process is not clear in x-ray films. The medial branch runs around the lateral border of the articular process at the root of the transverse process and is attached to the periosteum by connective tissue. Thus, the junction of the superior articular process and the root of the transverse process are commonly targeted for the block and neurotomy of the medial branch.

The distance between the root of the transverse process/the midpoint of the fibro-osseous canal and the posterior median line/body surface is a useful reference for the needle position and depth of percutaneous treatment. We found that, when targeting the root of the transverse process, the entering point of the needle should be ∼2.4 to 3.3 cm lateral to the median line at the level of the spinous process midpoint, with a needle depth of ∼3.1 to 4.6 cm. The lower lumbar vertebrae have increased values for the needle positioning. When the fibro-osseous canal is targeted, the entering point of the needle should be ∼2.1 to 2.9 cm lateral to the median line, with a needle depth of ∼2.2 to 4.0 cm. Also, the lower lumbar vertebrae have increased values for the needle positioning for this condition. The dorsal ramus is accompanied by vessels and repeated puncture may cause bleeding and hematoma. We found that the distance between the dorsal ramus bifurcation and the superior border of the root of the transverse process is ∼3 mm. Therefore, puncture at 3 mm superior to the root of the transverse process involves high risk of injuring the dorsal ramus. In addition, the dorsal ramus is only 1 cm from the ventral ramus. Thus deeper puncture should be avoided in the case of injury to the nerves and vessels.

The facet joint is innervated by the medial branch of the dorsal rami from 2 adjacent spinal segments. The nerve fibers of the medial and the lateral branches may arise from the adjacent 2 to 3 spinal segments. Therefore, usually 2 to 3 adjacent spinal segments are involved in the block and neurotomy of the medial branch. For example, low back pain caused by the L4–5 facet joints is treated by blocking the medial branches of the L3–4 dorsal rami. The multifidus muscles are critical for the spinal stability and are innervated solely by the medial branch. The medial branches run on the deep aspect of the multifidus muscles, and each muscle bundle is solely supplied by 1 branch, without communicating braches.^18,19^ Neurotomy of excessive medial branches may cause denervation of the multifidus muscles and spinal instability. We recommend avoiding neurotomy of the medial branches of 3 or more spinal segments.

## CONCLUSIONS

The lumbar facet joint is innervated by the medial branch of the spinal dorsal ramus. For the percutaneous treatment of block and neurotomy, the first choice of target is the medial branch fibro-osseous canal near to the accessory process. The entering point of the needle is ∼2.1 to 2.9 cm lateral to the median line, with a needle depth of ∼2.2 to 4.0 cm. The accessory process is not displayed in x-ray films; therefore the junction of the superior articular process and the root of the transverse process are targeted. The entering point of the needle is ∼2.4 to 3.3 cm lateral to the median line, with a needle depth of ∼3.1 to 4.6 cm.

## References

[1] Dreyfuss PH Dreyer SJ Herring SA Lumbar zygapophysial (facet) joint injections. *Spine* 1995; 20:2040–2047. 8578383 10.1097/00007632-199509150-00019

[2] Takahashi Y Ohtori S Takahashi K Dorsoventral organization of sensory nerves in the lumbar spine as indicated by double labeling of dorsal root ganglion neurons. *J Orthop Sci* 2010; 15:578–583. 20721728 10.1007/s00776-010-1482-0

[3] Ashton IK Ashton BA Gibson SJ Morphological basis for back pain: the demonstration of nerve fibers and neuropeptides in the lumbar facet joint capsule but not in ligamentum flavum. *J Orthop Res* 1992; 10:72–78. 1530799 10.1002/jor.1100100109

[4] Lakemeier S Lind M Schultz W A comparison of intraarticular lumbar facet joint steroid injections and lumbar facet joint radiofrequency denervation in the treatment of low back pain: a randomized, controlled, double-blind trial. *Anesth Analg* 2013; 117:228–235. 23632051 10.1213/ANE.0b013e3182910c4d

[5] Burnham RS Holitski S Dinu I A prospective outcome study on the effects of facet joint radiofrequency denervation on pain, analgesic intake, disability, satisfaction, cost, and employment. *Arch Phys Med Rehabil* 2009; 90:201–205. 19236974 10.1016/j.apmr.2008.07.021

[6] Gofeld M Jitendra J Faclier G Radiofrequency denervation of the lumbar zygapophysial joints: 10-year prospective clinical audit. *Pain Physician* 2007; 10:291–300. 17387351

[7] Gofeld M Faclier G Radiofrequency denervation of the lumbar zygapophysial joints—targeting the best practice. *Pain Med* 2008; 9:204–211. 18298703 10.1111/j.1526-4637.2007.00345.x

[8] Bogduk N Long DM The anatomy of the so-called “articular nerves” and their relationship to facet denervation in the treatment of low-back pain. *J Neurosurg* 1979; 51:172–177. 156249 10.3171/jns.1979.51.2.0172

[9] Bogduk N Wilson AS Tynan W The human lumbar dorsal rami. *J Anat* 1982; 134 (Pt 2):383–397. 7076562 PMC1167925

[10] Demondion X Vidal C Glaude E The posterior lumbar ramus: CT-anatomic correlation and propositions of new sites of infiltration. *AJNR Am J Neuroradiol* 2005; 26:706–710. 15814909 PMC7977091

[11] Sehgal N Shah RV McKenzie-Brown AM Diagnostic utility of facet (zygapophysial) joint injections in chronic spinal pain: a systematic review of evidence. *Pain Physician* 2005; 8:211–224. 16850075

[12] Bogduk N The innervation of the lumbar spine. *Spine* 1983; 8:286–293. 6226119 10.1097/00007632-198304000-00009

[13] Bradley KC The anatomy of backache. *Aust N Z J Surg* 1974; 44:227–232. 4282245 10.1111/j.1445-2197.1974.tb04409.x

[14] Masala S Nano G Mammucari M Medial branch neurotomy in low back pain. *Neuroradiology* 2012; 54:737–744. 22006423 10.1007/s00234-011-0968-6

[15] Yilmaz C Kabatas S Cansever T Radiofrequency facet joint neurotomy in treatment of facet syndrome. *J Spinal Disord Tech* 2010; 23:480–485. 20124916 10.1097/BSD.0b013e3181bf1c76

[16] Wolter T Deininger M Hubbe U Cryoneurolysis for zygapophyseal joint pain: a retrospective analysis of 117 interventions. *Acta Neurochir (Wien)* 2011; 153:1011–1019. 21359539 10.1007/s00701-011-0966-9

[17] Boswell MV Colson JD Sehgal N A systematic review of therapeutic facet joint interventions in chronic spinal pain. *Pain Physician* 2007; 10:229–253. 17256032

[18] Hansen L de Zee M Rasmussen J Anatomy and biomechanics of the back muscles in the lumbar spine with reference to biomechanical modeling. *Spine* 2006; 31:1888–1899. 16924205 10.1097/01.brs.0000229232.66090.58

[19] Bogduk N A reappraisal of the anatomy of the human lumbar erector spinae. *J Anat* 1980; 131 (Pt 3):525–540. 7216917 PMC1233250

